# Influenza A virus survival in water is influenced by the origin species of the host cell

**DOI:** 10.1111/irv.12179

**Published:** 2013-09-23

**Authors:** Sayuri Shigematsu, Amélie Dublineau, Olivier Sawoo, Christophe Batéjat, Toshifumi Matsuyama, India Leclercq, Jean-Claude Manuguerra

**Affiliations:** a Institut Pasteur, Environment and Infectious risks Unit, Laboratory for Urgent Response to Biological Threats (CIBU)Paris, France; b Nagasaki University Graduate School of Biomedical Science, Division of Cytokine Signaling, Department of Molecular Microbiology and ImmunologyNagasaki, Japan; c University of Paris Diderot, Sorbonne Paris Cité (Cellule Pasteur)Paris, France

**Keywords:** Host cell, influenza, lipid bilayer, survival

## Abstract

**Background:**

Influenza A viruses have an envelope made of a lipid bilayer and two surface glycoproteins, the hemagglutinin and the neuraminidase. The structure of the virus is directly dependent on the genetic makeup of the viral genome except the glycosylation moieties and the composition of the lipid bilayer. They both depend on the host cell and are in direct contact with the environment, such as air or water. Virus survival is important for virus transmission from contaminated waters in the case of wild aquatic birds or from contaminated surface or air for humans.

**Objective:**

The objective of this study was to check whether the origin species of the host cell has an influence on influenza A virus survival.

**Method:**

The persistence in water at 35°C of viruses grown on either mammalian cells or avian cells and belonging to two different subtypes H1N1 and H5N1 was compared.

**Results:**

Both H5N1 and H1N1 viruses remained infectious for periods of time as long as 19–25 days, respectively. However, within the same subtype, viruses grown on mammalian cells were more stable in water at 35°C than their counterparts grown on avian cells, even for viruses sharing the same genetic background.

**Conclusions:**

This difference in virus stability outside the host is probably connected to the nature of the lipid bilayer taken from the cell or to the carbohydrate side chains of the virus surface glycoproteins. Moreover, the long-lasting survival time might have a critical role in the ecology of influenza viruses, especially for avian viruses.

## Introduction

Influenza A viruses are major pathogens for an array of avian and mammalian species, including humans. Indeed, wild aquatic birds are the reservoir of the widest diversity of influenza A viruses, which can undergo genetic events associated with species barrier crossing. The role of the environment in the ecology of avian influenza viruses is not well understood. These segmented single-stranded negative RNA viruses have an envelope and viral proteins including two surface glycoproteins, the hemagglutinin (HA) and the neuraminidase (NA).^1^ The structure of the virus is directly dependent on the genetic makeup of the viral genome except the glycosylation moieties of the HA and the NA, and the composition of the lipid bilayer. They both depend on the host cell. Species-specific differences in the structures of oligosaccharides of glycoproteins have been well documented.^2^ Lipid composition varies with species, age, and physiological status of the cells.^3^ Glycerophospholipids, sphingolipids, and cholesterol are major components of the cell lipid bilayer of vertebrates.^4^,^5^ The sphingolipids, formed of ceramide, sphingomyelin, and glycosphingolipids (GSLs), are involved in many biologically important phenomena, involving cell polarity, membrane dynamics and cell–cell and cell–ligand interactions.^6^ The content and pattern of gangliosides, derived from GSLs, of several species and different organs demonstrated variability among species.^7^–^12^ For example, GM3 ganglioside was found as a major component in ten animal species, such as humans, chicken, dog, monkey, and rabbit, but there were significant variations in the distribution of minor gangliosides.^10^ Moreover, differences may exist among tissues in a specific species.^8^ The cholesterol, an essential component of the lipid bilayer, is integrated between the phospholipids. Through the interaction with the phospholipids and the sphingolipids, it modifies the physical, structural, and dynamic properties of membranes.^13^ Thus, the fluidity of the bilayer is influenced by the lipid and carbohydrate contents of the plasma membrane. Influenza viruses utilize lipid raft domains in the plasma membrane of infected cells as sites of virus assembly and budding. Lipid rafts, selected by influenza viruses during budding from plasma membrane, are variably sized and rich in cholesterol and sphingolipids.^14^,^15^ HA and NA are intrinsically associated with lipid raft domains, whereas the M2 protein is excluded from these domains.^16^ In virions, lipid bilayers are in direct contact with the environment, such as air or water. We have shown previously that the 2009 H1N1 influenza virus can persist outside the host for as long as 3 years, depending on the tested parameters. Furthermore, our experiments suggested that external structures, more directly in contact with the environment than genomic RNA, are mostly involved in virus loss of infectivity.^17^ Until now, only one study described a molecular determinant of resistance of influenza A viruses in natural reservoirs and systems.^18^ To verify whether the origin species of the host cell has an influence on influenza A virus survival, we compared the persistence in water of viruses grown on either mammalian cells or avian cells and belonging to two different subtypes H1N1 and H5N1.

## Materials and methods

### Cells and viruses

Madin Darby canine kidney cells were maintained in minimum essential medium (MEM 1X; GIBCO, Invitrogen, Carlsbad, CA, USA), supplemented with 10% fetal calf serum (FCS), antibiotics (0·1 units penicillin, 0·1 μg streptomycin/ml, GIBCO, Invitrogen), and tricine (10 mm; Sigma, Saint Louis, MO, USA) at 37°C in humidified 5% CO_2_ incubator. *Coturnix coturnix japonica* fibroblasts (QT6) were grown in Ham-F10 medium (F10 1X; Invitrogen) supplemented with 10% heat-inactivated FCS, 1% heat-inactivated chicken serum, 0·1 units penicillin, 0·1 μg/ml streptomycin, and 2% tryptose phosphate broth (Eurobio, Les Ulis, France).

Seasonal influenza A/New Caledonia/20/99 (H1N1) and influenza A/Hong Kong/156/97 (H5N1) strains were grown on either MDCK cells or QT6 cells, after propagation in embryonated chicken eggs followed by serial passages on MDCK cells (passage history described in Table 1). The fifth- and sixth-passage viral stocks were prepared as follows: monolayers of MDCK or QT6 cells in 75-cm^2^ tissue culture flasks were washed twice with 1X Dulbecco's phosphate-buffered saline (DPBS; GIBCO, Invitrogen) and then inoculated with 800 μl of initial virus suspension diluted in FCS-free MEM or FCS-free F10 respectively to a multiplicity of infection (m.o.i) of 0·01. After 1 hour adsorption at 35°C in 5% CO_2_, the inoculum was removed and cells washed twice with 1X DPBS. FCS-free MEM containing 2 μg/ml trypsin-TPCK or FCS-free F10 medium containing 0·4 μg/ml was added to MDCK and QT6 cells, respectively. The cell supernatants were collected 3 days post-infection and centrifuged to remove cell debris. Progeny viruses were stocked at −80°C.

**Table 1:** Passage history of H1N1 and H5N1 viruses. Both viruses were passaged on embryonated chicken eggs (w) and next five times on cell culture (c), either on Madin Darby canine kidney (MDCK) cells or on QT6 cells. (*) Virus preparations used in survival kinetics

	Passage history						Initial titer (TCID_50_ per ml)
	
	1st w	2nd wc1	3rd wc2	4th wc3	5th wc4	6th wc5*	
Virus designation							
H1N1 + M	Egg	MDCK	MDCK	MDCK	MDCK	MDCK	10^8·1^
H1N1 + Q	Egg	MDCK	MDCK	MDCK	MDCK	QT6	10^5·44^
H5N1 + M	Egg	MDCK	MDCK	MDCK	MDCK	MDCK	10^8·27^
H5N1 + Q	Egg	MDCK	MDCK	MDCK	QT6	QT6	10^6·58^

### Titration by endpoint method

Viral infectivity was estimated using endpoint titration, and tissue culture infectious dose 50 per ml (TCID_50_ per ml) value was calculated according to Reed and Muench's method.^19^ For endpoint titration, 3·3 × 10^4^ MDCK cells were seeded on each well of a microtiter 96-well plate. After 24–36 hours, subconfluent monolayers of MDCK cells were washed twice with 1X DPBS, and FCS-free medium was added and left until infection. Cells were infected as described previously,^17^ except eight-row replicates instead of four were performed for each sample. Examination for cytopathic effects was performed with light microscopy. The minimal detectable limit of this assay was 10^1·57^ TCID_50_ per ml.

The m.o.i was determined in plaque-forming unit (PFU)/ml based on the following equation: (log titer of TCID_50_ per ml) * 0·7 = titer PFU/ml.^20^

### Real-time RT-PCR

Viral RNAs were extracted using the NucleoSpin 96 Virus kit (Macherey-Nagel, Duren, Germany) according to the manufacturer's instructions. Extracted vRNAs were kept at −80°C for long-term storage and at −20°C for short-term storage. qRT-PCR targeting the M gene was carried out using primers and probes developed by the French National Influenza Reference Centers with a Light Cycler 480 instrument (Roche, Boulogne-Billancourt, France) and a SuperScript III Platinum OneStep RT-PCR kit (Invitrogen). For the HA segment, two qRT-PCRs using TTCCTTAATGTGCCAGAATGGTCTT and GGTTTGTACTGCTCAATAGGTGTTTC as primers for the beginning of the gene (120 pb) and GAGAGGAAATAAGTGGAGTAAAATTGGA and AAGATAGACCAGCTACCATGATTGC as primers for the end of the gene (110 pb) were performed. Both reactions were performed using the LightCycler® RNA Amplification Kit SYBR Green (Roche) in a 20-μl reaction mix [4 μl of LightCycler RT-PCR reaction mix SYBR Green I, 2·4 μl MgCl_2_, 2 μl resolution solution, 0·3 μl of primer mix (each 0·375 μm final) for the qRT-PCR amplifying the beginning of the HA gene or 0·4 μl primer mix (each 0·5 μm final) for the qRT-PCR amplifying the end of the gene, 0·4 μl LightCycler RT-PCR enzyme mix, 5 μl of template RNA] with a LightCycler® 480 Instrument. The final mix was submitted to the following steps: reverse transcription (55°C for 30 minutes), denaturation (95°C for 30 seconds), and 50 cycles of amplification (95°C for 5 seconds, 50°C or 53°C for qRT-PCR amplifying the beginning or the end of the gene, respectively, for 30 seconds, and 72°C for 7 seconds).

### Endpoint RT-PCR

RT-PCR targeting the M and HA genes was performed in two steps. Reverse transcription was carried out using SuperScript III First-Strand kit (Invitrogen) and primer annealing in the untranslated genomic region. PCR was performed with MPBio Taq CORE kit (MP Bio, Santa Ana, CA, USA) or PFU Turbo PCR kit (Agilent, Santa Clara, CA, USA). Sequences of the primers and probes used in the qRT-PCR and the two-step endpoint RT-PCR are the same as our precedent work for the M gene.^17^ Forward AAATGGAGAAAACAGTGCTT and reverse CAAATTCTGCATTGTAACGA primers were used for the amplification of the HA gene.

### Experimental procedure for trials in water

The kinetics of viral survival in water was carried out as previously described,^17^ except for the following: three aliquots instead of two aliquots and eight-row instead of four-row replicate per aliquot were made for parallel titration.

### RNase treatment

H1N1 + M and H1N1 + Q strains were either exposed to a temperature of 35°C for 25 days or not, and the viral infectivity of each strain was assessed by endpoint titration (as described above). The viral strains were then treated with RNaseA/T1 Mix (Fermentas, Thermo Scientific, Waltham, MA, USA) for 25 minutes at 37°C. Comparison of the means was carried out by the paired Student's *t*-test.

H1N1 + M and H1N1 + Q strains were also treated with a 1·5% Triton X-100 non-ionic aqueous solution (Roche) for 25 minutes at room temperature before RNase treatment.

## Results

### Virus survival in water

**Figure 1:**
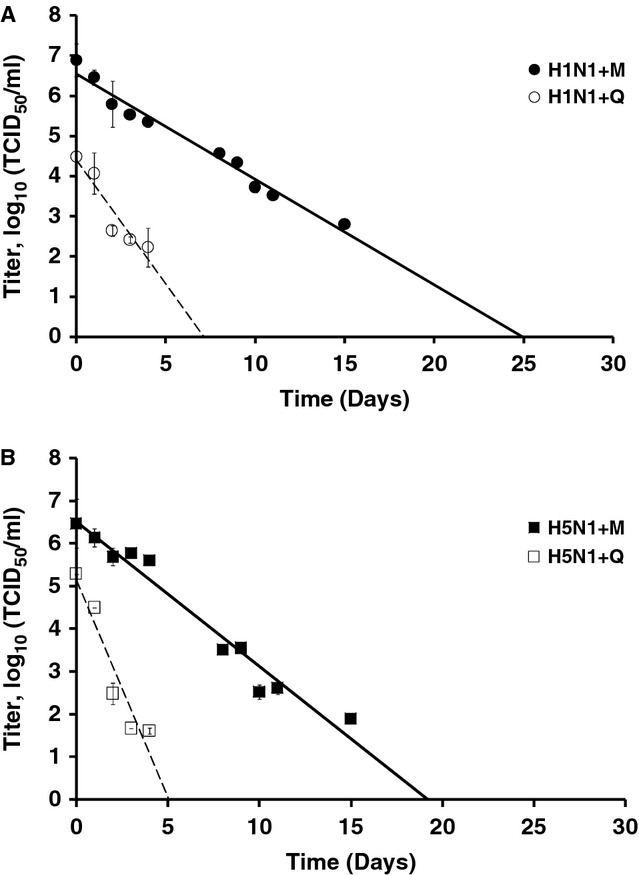
Virus survival in water at 35°C. Viral persistence of A/New Caledonia/20/99 (H1N1) (A) and A/Hong Kong/156/97 (H5N1) (B) grown on either Madin Darby canine kidney (—) or QT6 (····) cells in water at 35°C. TCID_50_ values corresponded with the mean values of the titers made in parallel from the three aliquots of water samples. Error bars represent the standard error of the mean.

The persistence in water of A/New Caledonia/20/99 (H1N1) and A/Hong Kong/156/97 (H5N1) derived from either mammalian Madin Darby canine kidney (MDCK; H1N1 + M or H5N1 + M) or avian (QT6; H1N1 + Q or H5N1 + Q) cells was evaluated at 35°C. Viral suspensions were first diluted 1:10 in distilled water and left for 30 minutes at the studied temperature. This short period of 30 minutes was arbitrarily chosen to determine the infectivity loss due to the initial change of environmental context for the virus. Microtiter endpoint titration was performed on MDCK cells as described in the ‘Materials and methods’ section, and the corresponding titer was called *T*_0_. There was no drastic loss of infectivity after 30 minutes for H5N1 and H1N1 viruses (1·80, 1·29, 1·21, and 0·95 log_10_ for H5N1 + M, H5N1 + Q, H1N1 + M, and H1N1 + Q, respectively) (data not shown). H1N1 and H5N1 strains were then left in distilled water for 35 days at 35°C. Aliquots were periodically removed to perform immediate endpoint titrations and determine TCID_50_ per ml values. Kinetics is represented in Figure 1, and TCID_50_ values corresponded to the mean values of the titers made in parallel for the three aliquots of infected water samples. For each condition, kinetics starts from *T*_0_. Linear regressions calculated from experimental values were obtained and used to determine the duration beyond which there was no more infectious virus, as previously described; 17 this duration corresponds with the *x*-intercept value (see Table 2). At 35°C, seasonal H1N1 virus strain propagated in MDCK cells (H1N1 + M) persisted for 25 days compared with 7 days when the same strain was propagated on QT6 cells (H1N1 + Q) (Figure 1A and Table 2). For the H5N1 strain, persistence estimates were 19 and 5 days when the virus was grown on MDCK (H5N1 + M) and QT6 (H5N1 + Q) cells, respectively (Figure 1B and Table 2). Because initial *T*_0_ was different for each growing condition (H1N1 + M, H1N1 + Q, H5N1 + M, and H5N1 + Q), we determined virucidy from the slopes calculated from linear regression. It corresponded to a reduction of 4 log_10_ of the titer according to the European Standards (NF EN 14476) (Table 2). No major differences in estimated virucidy between H1N1 and H5N1 strains were observed when both strains were amplified on the same cellular type: 6 versus 4 days for the H1N1 and H5N1 viruses grown on avian cells, respectively, and 15 versus 12 days for the H1N1 and H5N1 viruses grown on mammalian cells, respectively (Table 2). However, within a subtype, viruses with a mammalian lipid bilayer (H1N1 + M or H5N1 + M) survived longer than viruses with an avian lipid bilayer (H1N1 + Q or H5N1 + Q) at 35°C.

**Table 2:** Persistence times in water at 35°C. Slope (a), *y*-intercept (b), and *x*-intercept values of the linear regression straight line (log_10_ *y* = a*x* + b) calculated from experimental values. Virucidy corresponded to the duration necessary to obtain a fourfold reduction in the titer in log_10_

Strain	Temperature	*R*^2^	Lipid bilayer origin	Slope	*y*-intercept	*x*-intercept	Virucidy
				(a)	(b)	(days)	(days)
A/NewCaledonia/20/99 (H1N1)	35°C	0·97	Mammalian	−0·26	6·55	25 [23; 27]	15 [14; 16]
		0·89	Avian	−0·62	4·4	7 [6; 8]	6 [6; 7]
A/Hongkong/156/97 (H5N1)	35°C	0·96	Mammalian	−0·34	6·52	19 [18; 21]	12 [11; 13]
		0·91	Avian	−1·02	5·15	5 [4; 6]	4 [3; 5]

Persistence of multiple viral stocks, with different passage history, was also evaluated (Figure S1), and similar results were obtained, showing that differences persisted across trials.

### Quantification of viral genome

From each aliquot sample used to estimate TCID_50_ per ml values, viral RNA (vRNA) was also extracted to quantify genome copy numbers. Real-time RT-PCR targeting a small fragment (154 bp) of the M gene was performed as described in the ‘Materials and methods’ section. In Table 3, the genome copy number per ml for each experimental condition is listed. From d_0_ to d_35_, the RNA concentration (log_10_ copies/ml) varied between 10·36 log_10_ and 9·12 log_10_ for H1N1 + M, 10·84 log_10_ and 7·61 log_10_ for H1N1 + Q, 10·57 log_10_ and 9·3 log_10_ for H5N1 + M, and 10·35 log_10_ and 9·47 log_10_ for H5N1 + Q. RNA concentration was quite stable over time, whereas the infectivity decreased. In the same way, no variation in RNA concentration was obtained from real-time RT-PCR targeting two small regions of the HA segment (Table S1).

**Table 3:** M genomic segment concentrations. NA: not available. M genomic segment concentrations expressed in log(copy number per ml) obtained for H1N1 and H5N1 virus strains. Concentrations were determined at different days (designated as d*x*, *x* being the number of the day). RNA concentration at *d*_0_ was obtained after the viral suspension was left for 30 minutes in water at 35°C. All experiments were performed in triplicate

Virus designation	d_0_	d_1_	d_2_	d_4_	d_7_	d_8_	d_11_	d_15_	d_18_	d_22_	d_35_
H1N1 + M	10·37	10·17	10·28	10·02	9·76	9·89	9·69	9·63	9·45	9·44	9·23
	10·33	10·12	10·27	10·19	9·77	9·69	9·60	9·76	9·58	9·28	9·07
	10·39	10·19	10·09	9·95	9·72	9·75	9·80	9·59	9·53	9·42	9·05
H1N1 + Q	10·93	9·57	10·11	9·63	9·73	9·39	9·47	9·04	9·01	8·71	7·46
	10·76	9·83	9·92	9·67	9·52	9·42	9·10	8·94	8·79	8·67	7·46
	10·82	9·56	9·91	9·76	9·46	9·37	9·12	8·92	8·80	8·68	7·90
H5N1 + M	9·70	10·24	7·18	9·66	5·72	9·64	9·50	9·19	9·00	8·71	9·53
	11·06	11·16	6·00	9·84	9·71	10·05	9·52	9·32	9·04	8·99	9·24
	10·96	NA	10·55	10·41	NA	9·61	9·45	9·26	6·09	8·96	9·13
H5N1 + Q	10·61	10·56	10·58	10·12	9·99	9·99	9·86	9·84	9·95	9·55	8·51
	13·34	13·77	13·47	12·79	13·10	12·67	12·83	12·71	12·84	12·79	11·47
	10·11	10·38	10·37	10·04	9·99	9·82	9·89	9·77	9·96	9·29	8·44

### Integrity of the viral genome

**Figure 2:**
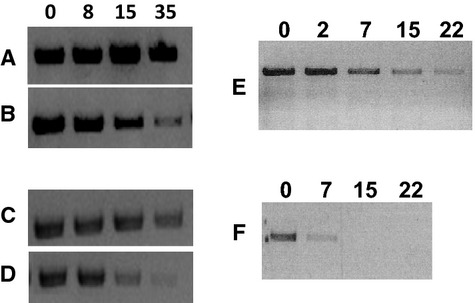
Endpoint RT-PCR targeting the whole M and hemagglutinin (HA) segments. RT-PCR targeting the M gene was performed for H1N1 (A, B) and H5N1 (C, D) viruses derived from Madin Darby canine kidney (MDCK) cells (A, C) or QT6 cells (B, D). RT-PCR targeting the HA gene was carried out on H5N1 viruses derived from MDCK cells (E) or QT6 cells (F). The indicated numbers corresponded to the time points of the kinetics (in days).

As the quantitative RT-PCR (qRT-PCR) assay targeted very small part of viral nucleic acid, genome integrity was also evaluated by using primers targeting the whole M segment. The 1027-bp RT-PCR product was detected on gel electrophoresis until day 35 for H1N1 and H5N1 viruses, grown on either MDCK cells or QT6 cells (Figure 2A–D). An endpoint RT-PCR targeting the whole HA gene was also performed, and the 1701-bp product could be detected until day 22 for H5N1 + M viruses and until day 7 for H5N1 + Q viruses (Figure 2E,F). These results suggested that a significant part of the viral genome was not degraded and remained intact in virus particles, even when infectious virus particles were not detected. Serial dilutions of the extracted RNA at day 0 and day 22 were carried out prior to RT-PCR targeting the full-length M or HA segments, for the H5N1 + M samples. Both genes were detected until day 22, even at a dilution of 1:100 for the HA gene (data not shown). These data reinforced our previous results suggesting that external viral structures more directly in contact with the medium than genomic RNA are mostly involved in loss of virus infectivity.^17^

### Integrity of the lipid bilayer

**Figure 3:**
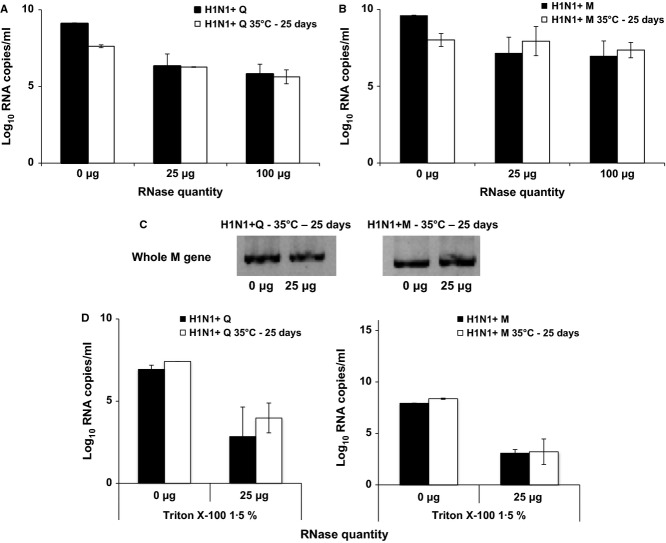
Evaluation of the lipid bilayer integrity. M genomic segment concentrations were determined for H1N1 viruses grown on QT6 (H1N1 + Q) (A) or Madin Darby canine kidney (H1N1 + M) cells (B) treated or not by RNase. H1N1 viruses were directly treated by RNase (▪: black bars) or put in water at 35°C for 25 days (□: white bars). (C) Endpoint RT-PCR was performed for H1N1 + Q and H1N1 + M viruses subjected to a temperature of 35°C for 25 days and then treated with RNase. (D) M genomic segment concentrations were determined for H1N1 + Q and H1N1 + M viruses treated by Triton X-100 1·5% prior to the RNase. Error bars corresponded to standard deviations (*n* = 3).

To explore that the loss of infectivity was not due to a mere viral burst during survival kinetics, water samples or viral stocks containing virus particles were submitted to RNase treatment in order to eliminate all non-enveloped genomes. First, the H1N1 + M and H1N1 + Q strains were submitted or not to a temperature of 35°C for 25 days, and endpoint titration was performed (data not shown). Each strain was then treated with different quantities of a mixture of RNase A and RNase T1 (0, 25, or 100 μg). After RNase treatment, the RNA concentration was quantified by the M gene qRT-PCR. For each quantity of RNase, RNA concentration was similar, whether the virus was exposed to 35°C during 25 days or not (all *P*-values were ≥0·4). However, differences were observed with genomes from QT6-derived viruses, which seem to be more sensitive to RNase treatment, when compared with the viruses grown on MDCK cells. After a 35°C kinetic and a 25 μg RNase treatment, RNA concentration decreased by 2 × 10^1^-fold for H1N1 + Q versus 5 × 10^−1^-fold for H1N1 + M (Figure 3A,B). The whole M segment was detected for both strains under each condition, showing that most part of the genome was protected (Figure 3C). These results were validated by an experiment in which the lipid bilayer was disrupted with a non-ionic detergent prior to RNase treatment. By exposing vRNA to RNase treatment, RNA concentration decreased by 1·8 × 10^3^- and 5·5 × 10^4^-fold for H1N1 + Q and H1N1 + M, respectively (Figure 3D). These results showed that a large part of the viral particles was not exploded at the end of the survival kinetics, emphasizing the fact that external viral structures are mostly involved in the loss of infectivity.

## Discussion

In this study, both seasonal H1N1 and H5N1 viruses remained infectious for periods of time as long as 1–3 weeks at a temperature as high as 35°C. These data are in line with our previous study about seasonal and pandemic H1N1 viruses. The stability of viral genome concentration and the existence of full-length genomic segments in the course of time confirmed previous data with different influenza strains, suggesting that internal structures are mostly not involved in the loss of virus infectivity.^17^ We showed that within the same subtype, human-origin viruses grown on mammalian cells were more stable than their counterparts grown on avian cells in water at 35°C. By the same way, a study highlighted that two isolates of an H2N3 avian-origin virus are more stable when grown on MDCK cells than in embryonated chicken eggs.^21^ Whole M genomic segment was detected even after treatment of viruses with RNase, suggesting that the detected genome was enveloped and that most of the viral particles were not exploded during the course of kinetics. Furthermore, in electron microscopy studies, no obvious differences, in term of integrity of the lipid bilayer (shape, diameter, protein density), were detected between influenza virus particles whether or not they were exposed to a given temperature for an extended period of time (data not shown).

Taken together, our data point out the role in virus survival outside the host of important external structures of the virions, which are not encoded by the viral genome: the lipid bilayer and the glycosylation moieties branched on the HA and the NA. Indeed, lipid bilayers, as well as the carbohydrate side chains of glycoproteins, are variable from species to species.^2^,^7^–^10^,^22^ Lipid membranes of MDCK cells were previously studied with differences observed between the apical and the basal membranes.^14^,^23^ A recent lipidomic analysis studying the lipid composition of the apical membrane of MDCK cells and of the envelope of influenza A viruses showed that the proportion of cholesterol and sphingolipids is enriched in the envelope of virions.^24^ The lipid composition of QT6 cells has not been described yet, but some studies on interspecies lipid analysis of various cells types and organs suggested differences between mammalian and avian cell bilayers.^25^,^26^ To point out these differences, the lipidomes of QT6 and MDCK cell bilayers are currently under analysis by gas chromatography–flame ionization detector. Budding of influenza particles occurs at the apical membrane of the infected cells, in specialized lipid domains called rafts to concentrate the viral components and to promote their interactions. These domains have distinct biochemical and biophysical properties in comparison with the rest of the plasma membrane.^27^,^28^ They stabilize oligomerized transmembrane proteins that can hence fulfill their biological function, like clustering of HA in rafts. Recently, lipid mobility studies by NMR showed that temperature-dependent lipids ordering domains in the viral membrane may also contribute to airborne transmission of viruses between individuals^29^ and thus may play an important role in the stability of the virus outside the host. Those findings may explain how the virus equipped with an envelope can independently, through changes in temperature, regulates its rigidity and therefore protection against the environment (cold temperatures) or helps its entry into a new host cell by promoting viral fusion (temperatures similar to natural infection).^30^ It has been previously shown that cholesterol-enriched lipid envelopes are more rigid and promote viral entry and infection.^31^ Our results, together with potential differences in cholesterol composition, could explain why human-origin viruses grown on avian cells are more susceptible to temperature than their counterparts grown on mammalian cells.

## Supporting Information

**Figure S1.** Virus survival in water at 35°C.

**Table S1.** HA genomic segment concentrations.
