# Supplementary material for: Influenza A virus survival in water is influenced by the origin species of the host cell
Source: Influenza Other Respir Viruses. 2013 Sep 23;8(1):123–30. doi: 10.1111/irv.12179 (PMC4177806; doi:10.1111/irv.12179)
Supplement: Supplementary file 1 — Figure S1. Virus survival in water at 35°C. [file irv0008-0123-SD1.docx]

**Figure S1**: Virus survival in water at 35°C. Virus persistence of A/Hong Kong/156/97 (H5N1) either grown on MDCK (—) or QT6 (····) cells with different passage histories. Numbers in subscript corresponded to the number of passages performed in each cell line. TCID_50_/mL values corresponded to the mean values of the titers made in parallel from the 3 aliquots of water samples. Error bars represent the standard error of the mean.
