# Supplementary material for: Influenza A virus survival in water is influenced by the origin species of the host cell
Source: Influenza Other Respir Viruses. 2013 Sep 23;8(1):123–30. doi: 10.1111/irv.12179 (PMC4177806; doi:10.1111/irv.12179)
Supplement: Supplementary file 2 — Table S1. HA genomic segment concentrations. [file irv0008-0123-SD2.docx]

| **Virus** | **Amplified region** | **d0** | | **d2** | **d7** | **d15** | **d22** |
| --- | --- | --- | --- | --- | --- | --- | --- |
| H5N1+M |  | 8.96 | 4.06 | | 8.84 | 7.79 | 7.73 |
|  | 284-404 | 9.05 | 8.44 | | 4.81 | 8.06 | 7.66 |
|  |  | 8.31 | 4.06 | | 5.73 | 8.02 | 7.10 |
|  |  | 8.98 | 6.77 | | 5.62 | 8.57 | 8.08 |
|  | 1560-1670 | 9.97 | 5.62 | | 9.11 | 8.63 | 8.21 |
|  |  | 9.86 | 9.68 | | 5.59 | 8.23 | 8.25 |
| H5N1+Q |  | 7.85 | 8.03 | | 7.90 | 6.57 | 7.20 |
|  | 284-404 | 8.04 | 8.16 | | 7.82 | 7.56 | 7.72 |
|  |  | 8.23 | 7.99 | | 7.78 | 7.57 | 7.53 |
|  |  | 9.11 | 9.17 | | 8.81 | 8.79 | 8.68 |
|  | 1560-1670 | 8.98 | 9.11 | | 8.88 | 8.73 | 8.85 |
|  |  | 8.86 | 9.02 | | 8.99 | 8.67 | 8.47 |

**Table 1S: HA genomic segment concentrations**. HA genomic segment concentrations expressed in log(copy number/mL) obtained for both H5N1 strains (H5N1+M and H5N1+Q) and for two different regions of the gene. Concentrations were determined for each triplicate at different days (designated as dx, x being the number of the day). RNA concentration at d0 was obtained after the viral suspension was left 30 min in water at 35°C.
